# Applying Positive Psychology to the L2 Classroom: Acknowledging and Fostering Emotions in L2 Writing

**DOI:** 10.3389/fpsyg.2022.925130

**Published:** 2022-08-03

**Authors:** David Byrd, Zsuzsanna Abrams

**Affiliations:** ^1^Department of Teacher Education, Weber State University, Ogden, UT, United States; ^2^Languages and Applied Linguistics, University of California, Santa Cruz, CA, United States

**Keywords:** L2 writing, EMPATHICS, emotions, positive psychology, L2 learning

## Abstract

The process of learning a new language can be filled with many emotions, both positive and negative, for the learner. This is particularly true in the area of writing, where students may feel a close connection to their sense of self. Thus far, the foreign language teaching profession has tended to prioritize cognition over emotion in research and classroom practice, with limited attention paid to the role of emotions in language learning. Recently, however, scholars, influenced by psychology, have taken a more active look at how emotions might mediate language learning. Among these scholars, Rebecca Oxford proposed a model that integrates tenets of positive psychology and second language learning, which she has designated as EMPATHICS. This nine-component model examines emotions/empathy, meaning/motivation, perseverance, agency/autonomy, time, hardiness/habits of mind, intelligences, characteristics, and self-factors. In this paper, we apply the EMPATHICS model to teaching second language writing and offer suggestions for task design at different stages of the writing process. While many second language teachers already incorporate some aspects of positive psychology in their classroom, becoming explicitly aware of its potential to foster better learning outcomes behooves us all.

## Introduction

Learning a second language (L2) can be an intense experience, where students are confronted with a myriad of new information: vocabulary, grammar, culture and writing and reading conventions that may differ greatly from those to which they are accustomed. Associated with these expectations come a variety of emotions that have an effect on how well individuals navigate learning the L2 (Oxford, 2016; Dewaele et al., 2019). While some emotions have been a mainstay of applied linguistics research (e.g., motivation and anxiety), more recently a new subfield has emerged that examines a broader view of how emotions mediate cognition. This subfield has embraced the tenets of positive psychology and seeks to understand how attending to learners’ emotions can help them meet the demands of learning other languages better.

Guiora (1983) suggests that language learning is sometimes seen as “a profoundly unsettling psychological proposition,” (p. 8) with feelings of insecurity and confusion (among others; see Pavlenko, 2006). Others (Marcos-Llindas and Juan Garau, 2009; Oxford, 2014) posit that even negative emotions can be used to help foster language learning. In a plenary address at the 2010 CLESOL conference in New Zealand, Swain (2013) talked about two poignant situations where profoundly negative emotions eventually led to great success in learning the L2, but success in spite of negative emotions is not easy, and one of the most emotion-wrought aspects of language learning can be writing, because as students write, they invest much of themselves and their sense of self into the written product (Casanave, 2007), be it in their first or another language. Learners often find the integrative task of L2 writing stressful, if they worry about their (perceived or real) lack of lexical, grammatical, pragmatic, rhetorical skills in the L2; they may struggle to transform their ideas into an L2 text, making them anxious about L2 writing tasks (Abdel Latif, 2015; Cheng, 2017).

Yet, due to the profession’s history of focusing on cognition over emotion, too often L2 teachers tend to ignore the emotional side of writing (Swain, 2013); they are too focused on accuracy and mechanics, even if content-based scores are included in their assessment. However, divorcing the emotions related to a given topic can lead to greater frustration, which, in turn, can influence the quality of the final product; conversely, acknowledging and supporting the experience of positive emotions can lead to long-term learning (both of writing and the L2 more generally), since emotions and cognition are deeply interwoven (Swain, 2013; Oxford, 2016; Butler, 2019; Dewaele and Li, 2020). Therefore, language instructors at all levels need to consider the connection between the cognitive aspect of writing in the L2 and the emotions related to the writing act. Fortunately, positive emotions can counterbalance negative ones that learners might have (Fredrickson, 2004). In the L2 writing context, this claim can be realized, if the opportunities presented by L2 writing—such as learners being able to express their thoughts in carefully scaffolded L2 writing tasks and demonstrate what they know, rather than what they have not yet mastered—and learners can develop more positive emotions about L2 writing. With this framing in mind, in this conceptual paper we discuss ways to incorporate tasks at different stages of the writing process to help teachers and students navigate the wild ride that is L2 writing. Specifically, we will connect L2 writing to positive psychology, the recognition that positive emotions play a key role in L2 teaching (Wang et al., 2021). It should be noted that much of what we do in teaching already relates to positive psychology practices, therefore we do not advocate for reinventing the metaphorical wheel of L2 writing pedagogy, but the discussion about emotions needs to be explicit.

## Literature Review

In Swain (2013) noted that “emotions are the elephant in the room—poorly studied, poorly understood, seen as inferior to rational thought” and are important in L2 learning and have been largely ignored by both researchers and practitioners (p. 195). She also posited that “the relationship between cognition is, minimally, interdependent; maximally, they are inseparable/integrated” (p. 196). This linkage has been long accepted in psychology. The “broaden-and-build” theory outlines how positive experiences in the moment—joy, interest, contentment and love—that are fostered through play, exploration and other enjoyable activities, help broaden an individual’s “momentary thought-action repertoires,” potentially generating not just immediate but also long-term physical, intellectual, social and psychological resources, such as support networks or resilience (Fredrickson, 2004, p. 1369). In the last decade, the tenets of positive psychology have made significant inroads in L2 pedagogy as well, although emotions still remain somewhat understudied in our field (MacIntyre and Gregersen, 2012; Oxford, 2016; Dewaele et al., 2019; Wang et al., 2021). Next, we briefly contextualize the profession’s move from a cognitive focus to its emergent embracing of positive psychology.

### L2 Teaching and Cognition

As suggested above, the L2 teaching profession has been primarily focused on cognition. The discipline’s roots in psychology and linguistics (behaviorism, cognitivism, structuralism) sidelined emotions (Swain, 2013). Quantitatively measurable performance tended to be the end goal of teaching a language, because it could be easily assessed; hence, research into learning or acquiring a language has been viewed as a cognitive science (Doughty and Long, 2003) and only recently has there been a sociocognitive shift that seeks to understand L2 learning more holistically, occurring both as an individual cognitive process and a socially contextualized, interpersonal one (Atkinson, 2013). A wide range of emotions, positive and negative, have a clear role in mental processes and merit further consideration.

### Positive Psychology

In Seligman and Csikszentmihalyi (2000) guest edited an issue of the journal *American Psychologist*, which was dedicated to articles discussing positive psychology, what it was and how it can influence people. The authors called for more attention to positive emotions and experiences, in order to help individuals thrive and flourish (*cf* Csikszentmihalyi and Nakamura, 2011). Positive psychology does not ignore or deny the existence of problems; rather, it complements challenges and the associated potential negative emotions with positive aspects counterpoints (Lopez and Snyder, 2016). It emphasizes teaching the whole person, tending to one’s affective as well as intellectual needs. Specifically, positive psychology seeks to “strengthen learners’ and teachers’ experiences of *flow, hope, courage, well-being, optimism, creativity, happiness, grit, resilience, strengths,* and *laughter* with the aim of enhancing learners’ linguistic progress” (Dewaele et al., 2019, p. 1, emphasis ours).

In the area of L2 teaching and learning, previous research had been done with affect (Dewaele et al., 2019), but this work focused on motivation and anxiety, without exploring positive emotions. Starting in the late 1990s, educational psychologists and teacher educators began work that looked at positive affect with the role of emotions in L2 learning (see Arnold, 1999; MacIntyre et al., 2009), but the field of positive psychology was still largely ignored in these early studies. In an innovative paper, MacIntyre and Gregersen (2012) were the first to highlight the importance of emotions in L2 learning, positing that they are semi-controllable and can influence students’ learning. They acknowledged that negative emotions exist but suggested that these could be reduced if teachers created safe environments in the classroom and harnessed positive emotions to create balance. When emotions are better balanced, these authors argued, students are in a better position to learn the L2 and build longer-term resiliency and hardiness. With a positive psychology lens, we can highlight what is best in each learner and foster their success and well-being in the L2 classroom. As teachers adapt the model we present for teaching writing, it is important to remember that most of these psychological features are not static, but rather dynamic constructs and therefore can be strengthened during positive psychology-based classroom practice.

## Applying Empathics to L2 Writing

Oxford (2016) proposed a pathway for integrating positive psychology and L2 learning, drawing attention to “important psychological forces that help learners achieve high well-being and progress rapidly, develop proficiency and relish the language learning experience” (p. 10). Her model helps explain why some students struggle in the same classrooms where others succeed. The acronym she uses—EMPHATICS—stands for emotion/empathy, meaning/motivation, perseverance, agency/autonomy, time, hardiness/habits of mind, intelligences, character strengths and self-factors (Oxford, 2016, p. 10). These emotional components create a dynamic system of advanced mental attributes and functions that are determined by a complex set of interconnected components that continually evolve over time (Dörnyei, 2009). While Oxford’s model is applicable to L2 learning and pedagogy more broadly, in this paper we focus specifically on ways in which components of EMPHATICS can be fostered while teaching L2 writing, as Table 1 illustrates.

**Table 1:** Mapping EMPATHICS onto L2 writing.

Emotion/empathy	Pre-writing
Meaning/motivation	Real-world, personal connection
Perseverance	Writing as process
Agency/autonomy	Choices of topics and expression, finding meaning in tasks
Time	Realistic time allocation
Hardiness/habits of mind	Commitment, control, challenge
Intelligences	Multimodal source and written texts
Characteristics	Self-reflective portfolio assessment
Self-factors	Reactions to feedback (self, peer and instructor), making plans

In the next sections, we briefly review each component of Oxford (2016) model and explain how it maps onto the L2 writing process.

### Emotion and Empathy

Cognition is inherently linked to emotion, and is not separate from it, since learners’ emotions, both positive and negative, mediate their attention and perception. What they pay attention to and how they understand the material presented to them is shaped in part to how interesting or difficult they find the content or specific tasks, for example. By tapping into learners’ interests—happiness, curiosity and joy—their learning experience can be broadened and strengthened, which helps learners approach new experiences positively, with less inhibition and anxiety (Fredrickson, 2004; Seligman, 2011). In addition to encouraging positive emotions, learners’ negative emotions, such as fear, insecurity or confusion also need to be acknowledged. Negative emotions can help build resilience, perseverance, anticipation and focus, if there is space in the classroom to examine and reflect upon them; learners can be guided to use humor to manage negative emotions, for example.

When planning a writing task, instructors can choose a broad topic that allows for various approaches to solving the task. During pre-writing activities learners could choose from a set of ideas within that topic that speaks to their feelings, both positive and negative. Brainstorming or debates can tap into learners’ positive emotions with engaging topics and opportunities for new learning (positive emotions), but they can also mitigate negative emotions by (a) sharing ideas with learners who may be struggling with the topic and (b) reviewing, activating or building relevant vocabulary, grammar, syntax or discourse modeling. This preparation helps reduce worries and anxiety (negative emotions) that learners often report feeling during the writing process (e.g., not knowing how to respond to prompts) and consequently builds learners’ confidence (Abrams and Byrd, 2016, 2017). Pre-writing activities such as interviews or primary source reading may also offer opportunities for learners to develop their ability to feel and express sympathy and compassion. These emotions, too, can be both positive (being happy for someone else) and negative (sharing someone else’s sorrow) and lead to empathy—an “other-oriented emotional response… [to the] perceived welfare of someone else” (Batson et al., 2011, p. 418). L2 learners can also learn to detach from the writing and acknowledge that they may not be completely prepared at this point to express their thoughts fully and tell themselves, perhaps using humor, to confront their fears.

**Figure 1:**
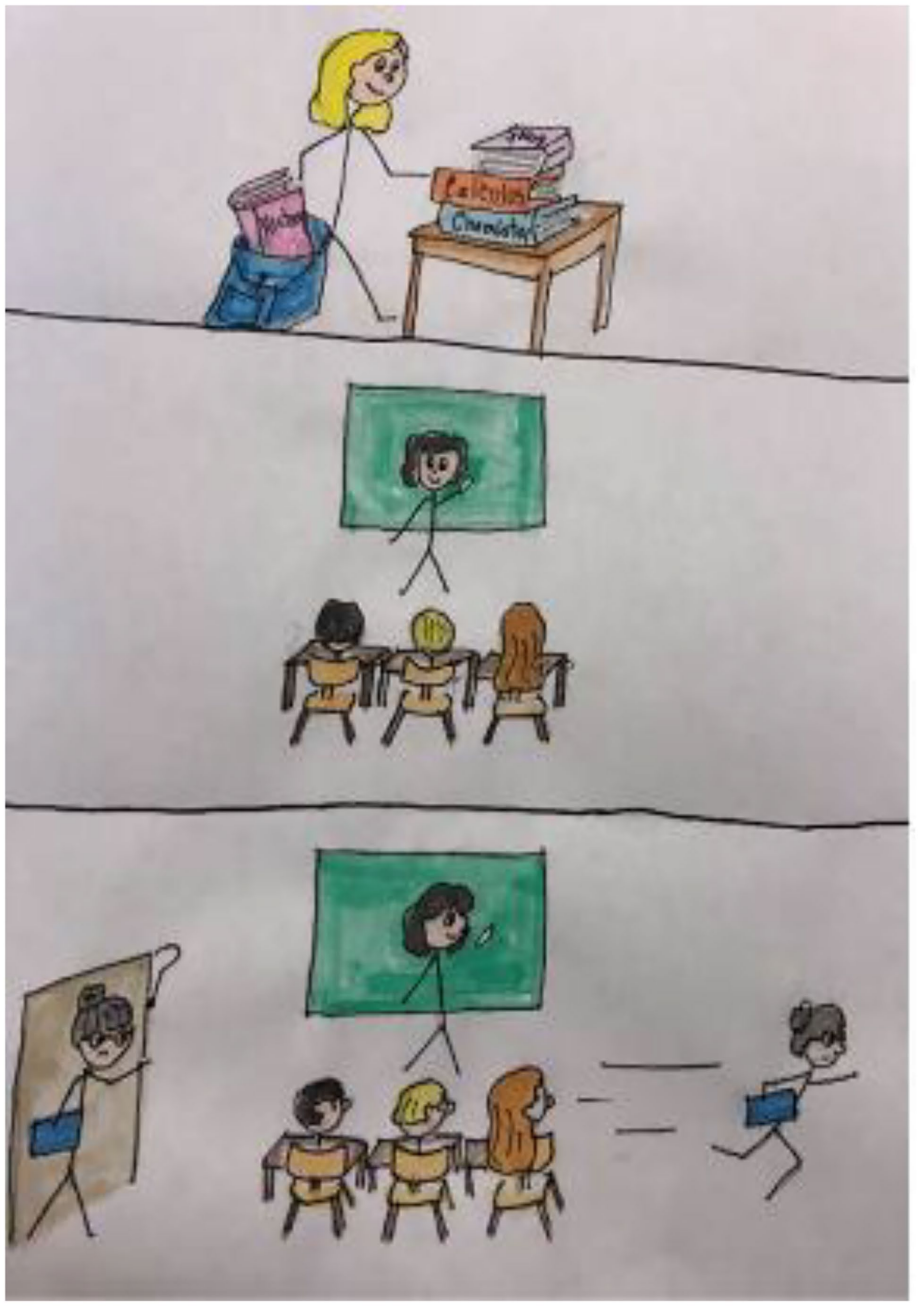
Example of a Drawing-based Pre-writing Task Reflecting Emotions: First day of Storyboard.

One possible pre-writing activity entails drawing something about the learner’s first days of school (see Figure 1), dream trip to another country or an influential person in their lives. In addition to the drawing, learners should describe their feelings (what was positive, negative), people who were present, and specific details pertaining to the topic (e.g., When was your first day of school? What was the scariest and most enjoyable about that day for you? What would you do during that trip? What would you be most excited about seeing or doing during that trip?). In order to build empathy, learners should describe the picture in a small group and ask questions about each other’s pictures:

With a partner, think about an influential person in your life.

- Take 1 min to draw a quick sketch of that person, avoid using any words at this time.
- Take 1 min per person to describe who that person is, how you know them, and why they are so influential in your life—add details that come to mind as you talk.
- Each listener should ask at least one question (add more details if your answer warrants it).
- De-brief in whole-group session (volunteers can present their drawings).

This type of activity is useful as a pre-writing task for expository writing. For other genres, such as argumentation, learners could focus on rhetorical stance as personal expression that allows them to tap into their emotions vis-a-vis various topics.^1^

### Meaning and Motivation

In the next component of EMPATHICS, Oxford (2016) considers meaning to be a guiding principle by which human experience is interpreted and organized, and defines it as “personal relevance and significance that give purpose to life” (p. 18). Successful tasks allow L2 learners to find personal meaning in the assignments and materials they interact with. Ideally, tasks lead to peak meaningful experiences that entail joy and sudden flashes of insights. During such moments of deep immersion in tasks, learners may experience “flow,” a “complete absorption in what one does,” arising out of a balanced pattern of growth between one’s skill level and the challenges posed by a task at hand (Nakamura and Csikszentmihalyi, 2016, p. 279).

Finding meaning in language learning is also linked to intrinsic motivation (Gardner’s integrative motivation), which is the desire to do something for its own sake, due to a sense of challenge, interest or enjoyment. In the L2 context, this might translate into the desire to learn another “language, traveling for fun, establishing meaningful relationships, striving for personal growth or contributing to social causes, without concerns regarding a particular outcome or external reward” (Abrams, 2020, p. 248). Research on L2 motivation distinguishes intrinsic from extrinsic motivation (Gardner’s instrumental motivation), which is viewed as “doing something as a means to an end” (Locke and Schattke, 2019, p. 277), such as learning a language in order to get a job or building relationships that will yield some professional benefit. A more recent model includes achievement motivation, which is an individual’s desire to do well on a task (e.g., doing well on a video project or a creative writing assignment), whether or not they find personal enjoyment in it or whether it helps them achieve larger goals (Klein et al., 2013). Importantly, each type of motivation can be equally conducive to language learning, and the type of motivation that drives a learner can change over time, when something that was originally external becomes personally valuable (Gardner, 2006; Locke and Schattke, 2019). For example, an individual may think of language learning only as fulfilling a requirement at first, but discovering that it is quite enjoyable and useful for meeting friends as time progresses. Oxford (2016) connects motivation to integrativeness, which she defines as a sincere personal interest in communicating with, taking on characteristics of and perhaps identifying with members of another culture, which requires a positive orientation toward the L2, its speakers and cultures, as well as toward specific learning situations.

Well-designed language learning activities should prompt reflection allowing learners to notice progress, thus promoting their own recognition and discovery of meaning and motivation from the writing task. A metacognitive activity, for example, would entail keeping a learning journal, not primarily to practice vocabulary or grammar but rather to explore why a topic or a task is important for the learner or useful to cover. One such useful metacognitive activity would be a formative mid-term course evaluation that allows the instructor to find out what topics or tasks students find particularly (de)motivating and optimally challenging. Similarly, allowing learners to pick the genre that they want to use on an assignment can help increase their motivation, as can assigning tasks that give them an opportunity to connect the learning process to their own contexts. For example, they can interview a grandparent, a fellow student who is learning the L2 or a more advanced learner who’s overcome struggles with the L2. Likewise, students could interview peers who have studied abroad or native speakers, perhaps as invited guests to be interviewed in class. Learners should pre-write questions, take notes, and write an interview-based report (e.g., journal article) to explore topics relating to the cultures where the L2 is spoken. Such interactions can help learners tap into sources of both intrinsic and extrinsic motivation, while creative writing tasks can help foster achievement motivation.

### Perseverance and Resilience

Oxford (2016) describes perseverance as a “continued effort to do or achieve something despite difficulties, failure or opposition;” while it entails hope and optimism, one of the most salient components of perseverance is resilience (p. 29). Importantly, resilience is the “self-righting capacity” individuals have that they use to adapt to new situations, even in “the face of trauma, adversity, and/or everyday stress,” which leads to increased perseverance (Truebridge, 2014, p. 12–13), and, generally speaking, “psychological fitness” (Seligman, 2011, p. 127). While overwhelming life events can diminish individual resilience, certain personality traits can help increase our ability to thrive despite challenges: being outgoing and adaptable to new situations, having a strong social support network, having clear goals and a sense of purpose, being able to find the meaning in life and specific tasks, having a sense of humor, and having a high level of emotional intelligence (Masten et al., 2011).

**Figure 2:**
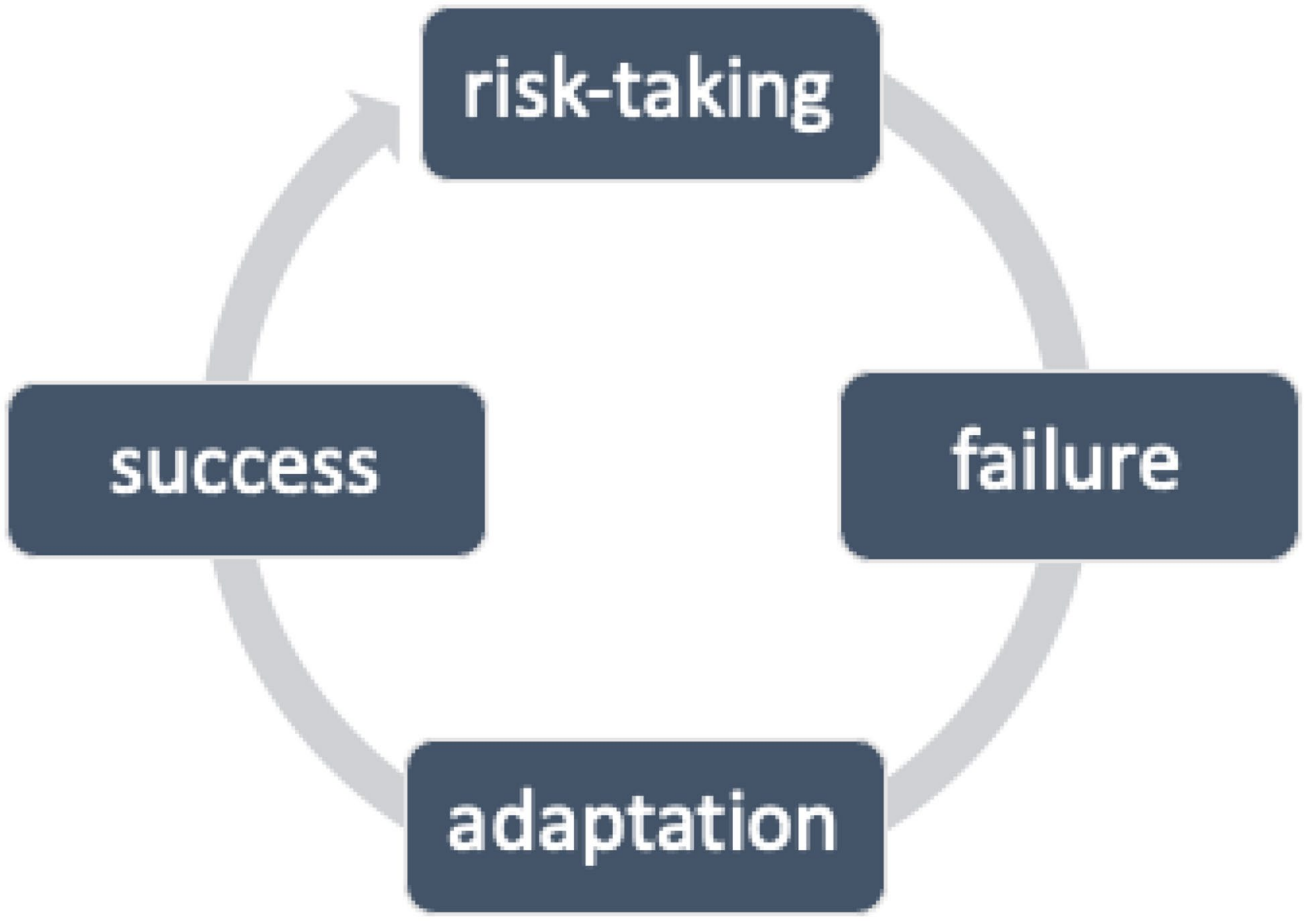
The Resilience Building Cycle (based on Oxford, 2016).

Resilience, as Oxford (2016) emphasizes, can be built and strengthened in a dynamic cycle of risk-taking → failure → adaptation → success (see Figure 2). In the L2 classroom, instructors can help learners develop resilience in several ways: structuring task sequences that allow learners to succeed (e.g., review relevant vocabulary, grammar, rhetorical structure, generate ideas prior to the main writing task); reading and reviewing good source text models learners can follow; providing opportunities for learners to pursue their own interests (e.g., select their own topic for the term paper); offering sufficient resources for learners to be able to complete tasks in a timely manner and with confidence; and acknowledging the learner as a whole person and orienting toward them with caring and compassion. Additionally, having learners share their work in smaller or larger groups can help them find out new ways of approaching content or tasks, which allows them to develop cognitive flexibility, further enhancing their resilience. Once learners submit their written work, instructors can support them by providing feedback that focuses on the positive, not just the negative, highlighting learners’ strengths, not just their weaknesses. Ideally, the feedback focuses on fewer important mistakes that interfere with comprehensibility rather than addressing everything (especially items that are correct but the instructor would personally phrase differently). Research on feedback shows that targeted corrections are more effective for language development anyway (Reynolds and Kao, 2021; Villavicencio and Argudo, 2021), offering yet another area in which emotional and cognitive benefits go hand in hand. A couple of strategies may involve writing positive comments on one part of the paper (e.g., right margin) and needs-improvement comments on another (e.g., left margin), while grading online can be done with different colors or similar placements, if the platform allows.

By providing careful, thoughtful feedback in alignment with learners’ L2 developmental and affective needs, instructors can also help them set realistic goals for language learning, which is a key aspect of resilience in the sense that “hope is the cognitive expectation of goal attainment” (Oxford, 2016, p. 32). Additionally, revisions—which allow for the development of ideas as well as for deeper learning of vocabulary, structures, grammar and content—can ensure that learners see mistakes as just a natural part of learning that can be fixed through the recursivity of the writing process. Similarly, telling students about the instructor’s own experiences with L2 learning in humorous ways can help learners realize that mistakes are not tragic but rather an everyday part of L2 learning. Throughout the writing process, grading rubrics should reflect what learners have achieved, rather than merely deduct points for what is wrong. This can be achieved in part by phrasing the descriptors in positive ways. Moreover, possibly assigning a writing topic about a situation in which the learner successfully used the L2—or having them imagine what it would be like to live in a culture where they would use the L2 successfully (What would that feel and sound like?)—can bring about positive reflections on learners’ progress. Finally, when possible, instructors can reframe difficulties as challenges, include humorous stories as sources for L2 writing tasks, and allow students to choose their own source texts that they find particularly interesting, sufficiently challenging but attainable (nb: collaborative writing may help make challenging tasks, from reading the source texts to writing their own, more attainable for learners).

By implementing such teaching strategies, instructors can help learners gain resilience “assets” that Masten et al. (2011) describe as “human, social and material capital” (p. 120), and which foster perseverance in language learning.

### Agency and Autonomy

The next interrelated concepts in the EMPATHICS model are agency and autonomy. Gao and Zhang (2011) identify agency as a starting point for learner action; it may entail “capacity (ability), often adding will (deliberate choice), and occasionally mentioning intention, belief, knowledge, taking charge through action, and using pathways/strategies” (Oxford, 2017, p. 76). Agency is grounded in a learner’s sense of meaning, how “they interpret and interact with the perceived opportunities in their surroundings” (Mercer, 2015). In relation to L2 writing, agency might be encouraged, when learners find a task to be personally meaningful to their lives. Autonomy is closely related to agency (and the distinctions can be difficult to identify in some scholarship); in general terms, it describes the paths that learners might choose to take—and the control they might have—to complete tasks their own way. Gao (personal communication; cited in Huang and Benson, 2013) considers agency as a universal (the ability of a person to make decisions) and autonomy as dependent on social mediation and the constraints of particular contexts. Oxford (2016) emphasizes that a sense of agency is a significant step in achieving autonomy. The former prompts learners to see that they have the ability to achieve goals they set for themselves, while the latter is a frame of mind that enables individual learners to create meaning around a task, for example, and take charge of their own learning (e.g., choose a plan of action, select priorities). Huang (2011) captures the distinction as follows: “agency [can be understood as] “entailing actions arising from deliberation and choice,” and autonomy “the capacity to do” (p. 242). Oxford (2016), cautions us though that both agency and autonomy are culturally dependent phenomena; that is, cultures have different notions and realizations of autonomy. In the L2 context, agency and autonomy can be best described as a dynamic process of gaining independence in both L2 learning (the process) and L2 use, and are directly connected to strategies that can be taught to promote their development in order to further learning.

In a study of 55 Swedish university students in an English language teacher training program, Sauro and Sundmark (2016) designed a task-based, computer-supported, collaborative, multimedia writing project, in which participants, working in small groups, had to create role-playing stories based on Tolkien’s *The Hobbit* (using filmic resources as well). The fan-fiction genre was particularly well-suited for the project, because it allowed learners to draft, reflect, generate new content and build a story around “a missing moment” from *The Hobbit* (p. 416), from one character’s perspective; while the overarching story was stipulated by the instructors/researchers, the groups had full autonomy “at the level of plot choice, rating, theme, character selection, literary devices used, and point of view” (p. 417). The project, as the authors relate the findings, encouraged students to analyze the source texts closely, reflect on their language choices over multiple iterative steps in the writing process, recognize and adopt genre-specific writing conventions, and implement creative writing techniques, all the while they were able to complete tasks that closely aligned with their language learning goals and personal interests.

As this study illustrates, giving learners options within larger tasks or projects encourages agency and autonomy, as choice allows them to achieve goals that are personally important to them (Ryan and Irie, 2014) and take charge of their own learning (Benson, 2011) even if only in small ways. Following a pre-writing brainstorming activity on any particular topic, learners could pick one to three different genres for which to develop the piece, unless a particular genre is required for specific learning outcomes. Potential options include expository writing, poem, limerick, graphic novel, dialog, song, interview, comedy sketch, newscast, movie scene, twitter thread (including or especially multimodal submissions), just to name a few ideas. Giving learners a choice of genre can help them find personal meaning in the task, while also encouraging freedom and responsibility for completing it. In order to get into the habit of creating more student-centered pedagogy, instructors should periodically ask themselves—and their students—what ideas they can try in order to provide students with more agency and autonomy in language learning.

Importantly, instructors have to recognize social autonomy (often referred to as Eastern style communal responsibility), not just Western-style individual autonomy (Kara, 2007). Social autonomy can be facilitated by taking a community-approach to decision-making (e.g., on topics, tasks, group assignments), discussing learners’ preference for valuing the community’s needs above one’s own, and recognizing that interdependence does not automatically mean that learners lack autonomy.

### Time and Selves

The amount of time language learning requires is significantly longer than most learners realize, both in terms of the general arc of L2 development and individual tasks. This little-discussed component of L2 learning merits honest discussion in the classroom, in particular the relationship between time, learners’ sense of self and L2 development. In L2 learning our past sense of self (self-view) influences how we see our present and future selves, multiple future selves, in fact (Dörnyei, 2009). A moderate orientation to the future seems to be associated with positive outcomes, because it increases motivation and planning, and facilitates delayed gratification and organization. Extreme future orientation, in contrast, is problematic, because it can lead to obsessive work toward a future goal, getting frustrated when that goal is not met, and feeling burnt out (while also not leaving time for pleasure, friendships and other pursuits outside of the classroom). Ideally, time-orientation is balanced, whereby learners attend to positive past selves (e.g., reflecting on goals they have been able to attain), present (e.g., monitoring progress toward goals learners set for themselves) and future (e.g., setting new goals as L2 development progresses) selves with flexibility.

In order to promote realistic expectations, two issues should be considered when designing writing tasks: timing and content. The former is fairly easy to implement: writing tasks should consist of multiple steps, including pre-writing idea generation, sufficient time for creating the first draft, providing feedback, allocating sufficient time for revisions, providing peer editing, and writing a final draft. In curricular planning, it is better to schedule fewer writing tasks with multiple steps than to have many isolated writing tasks. Time-related content considerations entail explicitly addressing what learners would like to learn and when, including discussions in small groups or as a class to brainstorm ideas and identify reasonable goals (e.g., How much can they actually learn in a ten-week term?). Although we often wish to learn as much and as quickly as possible, the reality is that L2 learning takes time and commitment, and learning it at the academic level can take as long as seven years (Cummins, 2021). To bring this issue to the forefront, the class could interview more advanced learners about their L2 experiences and write up the interview as a journal article, reflecting on the metacognitive aspects of L2 development. Alternatively, students could keep a learning journal, in which they identify personal goals, then check off each skill as they complete it, revising and setting future goals, acknowledging and celebrating accomplishments.

### Hardiness and Habits of Mind

As we noted, L2 learning takes time. It also takes commitment and consistency, as well as effective habits of mind. Commitment, drawing on motivation, can be defined as a learner’s willingness to “stick with” studying, even when they encounter challenges and difficulties, which are both a part of life and L2 learning. Costa and Kallick (2008) use the partial list of terms below as descriptors for habits of mind in their model:

persistence, flexibility, questioning and posing problems, applying existing knowledge to new situations, gathering information, persistence, openness to new ideas and experiences, engaging with others with humor, communicating clearly, and thinking independently.

Similarly, Oxford (2016) makes several recommendations for L2 learners to build effective habits of mind. Her recommendations include both individual, cognitively oriented components habits, such as creating plans for completing a task and finding effective strategies to fulfilling those plans (e.g., collaborating with a peer, asking others about how they solve language learning-related problems), ensuring a level of accuracy in the L2 to be able to communicate effectively with others, and reflecting on how the task is coming along and adjusting the plan of action if necessary to ensure task-completion. The habits also include interpersonal components: learning to listen to others with empathy and understanding and engaging with the L2 deeply by finding out about the cultures where the language is spoken (e.g., history or social/political/linguistic practices), which can help ensure that the learning process is meaningful. The final set of recommendations can be viewed as metacognitive strategies; they include finding out about how second languages are learned (e.g., by asking the instructor), because this information can help learners set reasonable expectations for their own L2 development, and thinking explicitly about previous language knowledge to facilitate language learning and language use (e.g., by identifying similarities between languages the learner already knows and their new language).

Helping learners control their reactions to challenges allows them to stay engaged instead of withdrawing from the learning process. One possible way for reframing the narrative is to encourage learners to view challenges as opportunities for growth, which in turn requires that L2 instructors give learners the chance to write creatively without penalizing them for trying out new knowledge and/or skills. When written texts include revisions, throughout the writing process, instructors can foster hardiness by giving learners opportunities to provide each other with support and care. Peer feedback guidelines should include a space for learners to identify strengths in other students’ work, and suggest places where their peers can elaborate, not just mark mistakes (Byrd, 2003). This approach requires training: learners need to receive explicit guidance on how to provide meaningful, constructive, useful feedback for their peers in a positive manner (e.g., “I really like the point you make here; it would be helpful if you could elaborate on this idea” or “While I understand your argument, it might be more effective if it were made earlier on in the paper”). When talking about positive feedback, it is also helpful to consider approaching mistakes with empathy; if learners can build empathy toward their peers’ mistakes, they may be more compassionate with themselves as well, which can further their willingness and ability to “stick with” L2 writing and L2 learning more generally.

As with time (see the previous section), an explicit, metacognitive discussion of both negative and positive habits of mind can be useful: What does it look like when learners withdraw? Why do they withdraw? What activity can be conducive to creativity? What are effective ways of maintaining control of the writing process? How can we give students more control (many students are dealing with mental and physical health issues) and still meet curricular demands?

### Intelligences

Positive psychology in L2 learning taps into multiple intelligences (*cf* Gardner, 1993, 2011), since each can help students improve their L2 skills, as Table 2 illustrates.

**Table 2:** Mapping multiple intelligences onto L2 Learning (based on and expanded from Oxford, 2016).

**Intelligences**	**L2 practices**
Bodily kinesthetic	Recognizing and producing/reciprocating gestures and body-language (e.g., bowing, the French shrug, Indian head-shake), proxemics (culturally and personally preferred closeness to others; Abrams, 2020)
Existential	Becoming aware of who we are as individuals, applying wisdom, compassion, integrity, joy, love, creativity, and peace to achieve a deeper sense of self (Singh Negi and Khanna, 2017); using spirituality to succeed in L2 learning and to connect to other cultures
Interpersonal	Establishing social connections to other users of the L2 (e.g., in physical or online communities), creating empathy toward others
Intrapersonal	Engaging actively in introspection, setting realistic goals for oneself and raising awareness surrounding issues of identity (who we were, are and who we wish to become)
Logical-mathematical	Recognizing patterns and rules, be it grammar, the lexicon or pronunciation
Musical	Recognizing and mimicking accent, pitch, intonation; especially important for learning tonal languages
Naturalistic	Connecting to local ecologies and natural environments, improving skills of intercultural communication (e.g., other cultures’ attitude toward and relationship to nature)
Verbal–linguistic	Interpreting and creating language in any modality (i.e., reading, writing, listening, speaking, learning components of sign-language)
Visual–spatial	Recognizing and producing characters, graphemes, symbols, organizing text, using sign-language

In addition to Gardner’s (1993, 2011) model, Sternberg (1985, 1997) considered practical intelligence (street smarts and real-world adaptability), creative intelligence (intuitively divergent responses to novel tasks; new ways of solving problems) and analytical intelligence (information processing and critical thinking skills that are testing on traditional intelligence tests) to be part of our repertoire. Around the same timeframe, Salovey and Mayer (1990) explored the concept of emotional intelligence, which refers to “the abilities to recognize and regulate emotions in ourselves and in others” (Goleman, 2003, p. 15).

As with previous components of the EMPATHICS model, applying intelligences to L2 writing has an indirect, metacognitive path and a direct one. Regarding the former, metacognitive path, it is helpful to draw learners’ attention to the fact that there are multiple intelligences involved in L2 learning. Many students come to us saying “I cannot learn languages,” because they had a bad experience previously. Reassuring them that there are many ways to learn a language can help alleviate some of their concerns, especially if they have an opportunity to reflect on their strengths and gain new skills with complementary intelligences (e.g., if they already have strong interpersonal intelligence, they can develop new strengths in verbal–linguistic intelligence). It might also be worthwhile to discuss different learning strategies that students might prefer—such as cognitive, metacognitive, affective and sociocultural interactive strategies (Oxford, 1990, 2011)—and expand their repertoire of strategies they can successfully implement in L2 learning.

In terms of the direct application of multiple intelligences to L2 learning, multimedia texts are ideal, as they tap into visual, textual, bodily, kinesthetic, auditory and other types of input sources; they can also serve as a springboard for output, as the following two tasks illustrate (Perveen, 2018). The first task entails analyzing how diversity is represented in the media. This task taps into (both drawing on and strengthening) interpersonal, bodily kinesthetic, analytic and emotional intelligence at minimum. Students at the intermediate or advanced level are asked to analyze images that represent people from diverse cultural backgrounds, examining how these images cultivate attitudes and beliefs about different groups of people, and reflect on how these images may promote stereotyping, identity and the rationalization of prejudice or privilege. After analyzing several media sources that the instructor can assign or learners might propose, students write a “white paper”—a report on the issue of media representation of cultural diversity, possibly including a recommended solution or action—either individually or in groups to encourage discussion and debate. The final product, along the lines of an investigative report we would see on television, would be a seven-slide multimodal project (PowerPoint presentation, Prezi or Adobe Spark), consisting of a title slide, five slides dedicated to the main subject and one slide for references. It must include written, visual (pictures, images, video clips, art), musical and spoken modalities that should enhance (at least, not distract from) each other and the entire project.

Another task similarly calls for both processing and producing multimodal content over a longer period of time. For this project, at the input stage, learners analyze the materials related to a television or streamed film series, watching the actual video (possibly with subtitles), reading the official summary and news releases, and exploring fan-fiction sites and social media reports by the main actors or the studio and the associated comments from viewers (e.g., Facebook, Twitter, Instagram). After this extended reading and viewing stage, learners can produce a variety of multimodal presentations: mock interviews with the main characters interspersed with videos, twitter threads with memes and other text-image combinations, fan fiction (just for the class or “live”), creative scripts based on the film (e.g., continuing the story or transposing it into a new context), a comedy sketch, just to name a few possibilities. Such tasks allow learners to tap into and enhance multiple intelligences, but also foster their L2 proficiency, build intercultural communicative competence, enhance their ability to empathize with others (e.g., by inhabiting other character roles, fictional or real), and improve an array of skills (e.g., study techniques, collaboration, problem-solving).

The use of creative, multimodal tasks requires rethinking from instructors, though, recognizing that today’s students need skills to consume and produce multimodal texts, defined in the broadest sense. A first step might be to reflect on a recent class activity we did with our students and redesign it so that it taps into and fosters at least two more types of intelligence than what it currently supports.

### Character Strengths

Character strengths relate to the previous components of the EMPATHICS Model, and overlap in particular with habits of mind, because “character strengths, like habits of mind, are habitual, deeply rooted and positive parts of the individual” (Oxford, 2016, p. 60). Oxford (2016) describes six categories of virtues, onto which character strengths can be mapped, as can be seen in Table 3.

**Table 3:** Categories of virtues (based on Oxford, 2016).

**Virtues**	**Character strengths**
Wisdom and knowledge	Creativity, curiosity, love of learning, open-mindedness, perspective
Courage	Authenticity, bravery, persistence, zest
Humanity	Kindness, love, social intelligence
Justice	Sense of fairness, leadership, teamwork
Temperance	Forgiveness/mercy, modesty/humility, prudence, self-regulation
Transcendence	Appreciation of beauty and excellence, gratitude, hope, humor, spirituality

These character strengths should bring personal fulfillment, satisfaction, and happiness, and should stem from personal values, rather than goal-oriented behaviors. The first set of character strengths, wisdom and knowledge, when applied to L2 learning, foster the use of creative approaches to studying, curiosity about another language and its related cultures, and seeing the world from other perspectives. Courage entails being responsible for one’s emotions and actions, meeting the demands of learning another language (e.g., extensive time) and the risks that might entail (e.g., not understanding everything), persevering despite these challenges, and even viewing them as an adventure. Humanity requires being kind to others during the learning journey: teachers, peers, and other users of the L2, tapping into one’s emotional intelligence. The fourth virtue, justice, should guide learners to remain fair and fight against biases toward members of other cultures, encouraging others to remain fair as well, possibly participating in projects or communities that promote intercultural understanding. Practicing forgiveness toward others, remaining modest and humble, taking considered risks, and reflecting on our own beliefs and behaviors belong to the virtue of temperance. The last set of character strengths fall under the rubric of transcendence and entail appreciating one’s own and other languages and cultures, expressing gratitude for the help of others (e.g., educators, more experienced L2 learners), remaining hopeful and optimistic, tapping into a sense of humor as we encounter mishaps during the L2 learning process, and finding meaning in life as an L2 learner.

**Figure 3:**
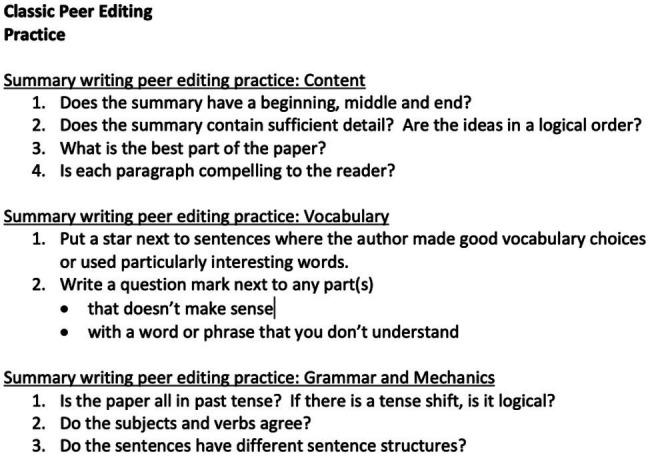
Guidelines for Peer editing to Increase Teamwork, Social Intelligence and Fairness (developed by DB).

**Figure 4:**
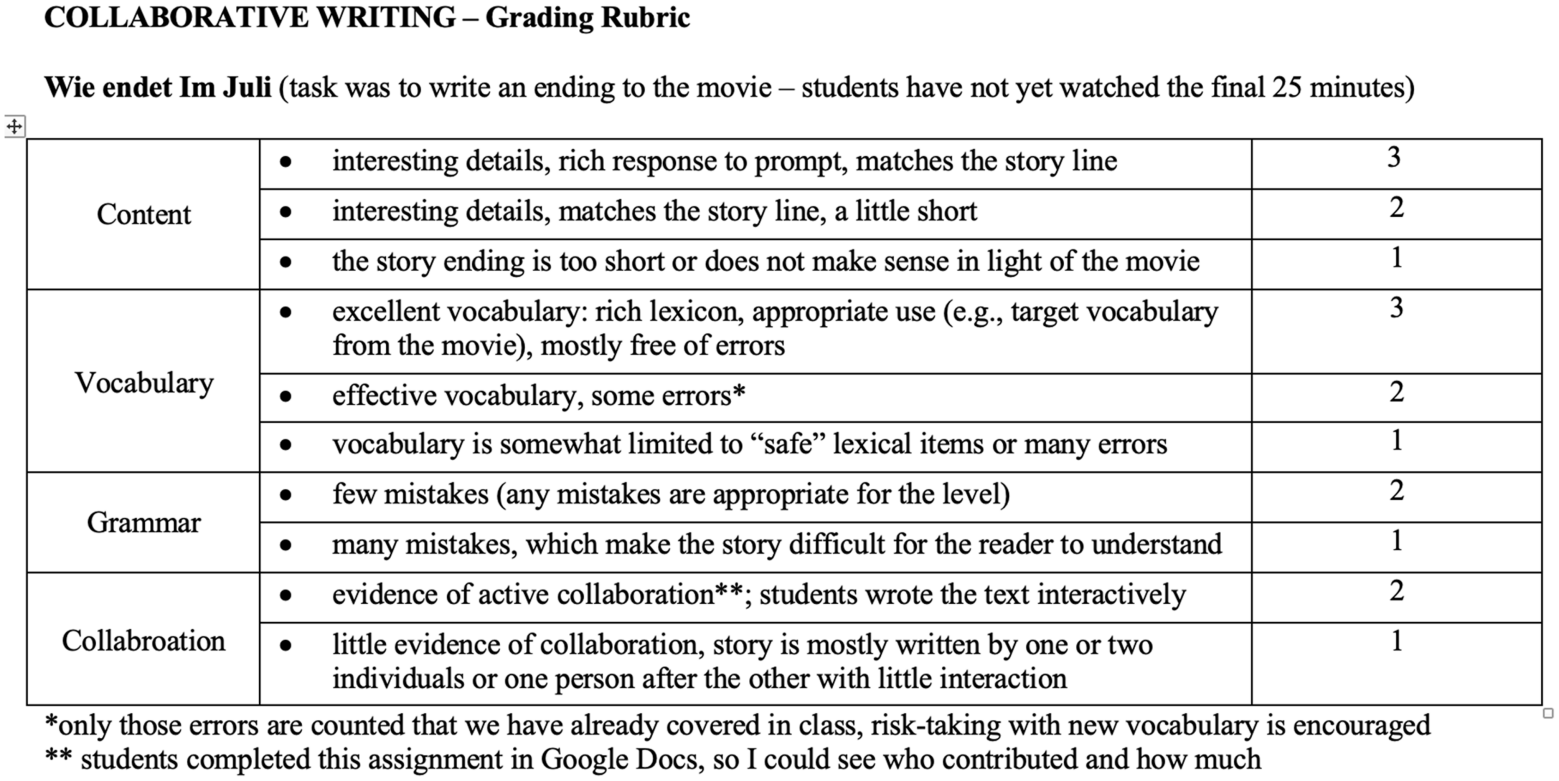
Holistic Rubric for Grading Collaborative Creative Writing Assignment (developed by ZsA).

These character strengths can be fostered during the writing process, for example at the editing and revision stages. The peer-editing process can be a positive experience in and of itself, if structured well, with clear instructions and goals, because they can model and emphasize building relationships of trust. The feedback guidelines should encourage positive feedback and positive comments, even where mistakes are present, but especially when the learner has written something interesting and/or creative, made a strong argument or offered useful details. In one peer review session, the class could try giving only positive comments on papers (see Hill, 2011). Moreover, letting learners see the grading criteria before they begin the writing process helps set clear expectations, mitigates stress, and eliminates unpleasant surprises. Working in specifics instead of generalities (e.g., “uses the simple past to narrate their weekend” instead of “uses appropriate verb tense”) helps provide much-needed clarity for novice writers. Two rubrics (see Figures 3, 4), developed and used by the authors, offer a few ideas for encouraging a positive approach to providing feedback on learners’ writing. As we implement peer- and teacher-feedback into writing, we should design task, editing and feedback guidelines that promote creativity, curiosity, the love of learning, open-mindedness, persistence, social intelligence, fairness, teamwork, appreciation, and humor (just to name a few character strengths mentioned above).

### Self-Factors

The final component of Oxford (2016) EMPATHICS model pertains to self-factors: self-efficacy, self-regulation, self-concept, self-esteem, and self-verification. The first of these concepts, self-efficacy, refers to confidence in our ability to accomplish a task, achieve goals we set and achieve (including the process of recognizing cause and [desirable] effect), and manage our behavior and environment. We gather information from our experiences (e.g., what kind of effort we exert, how and to what effect), others’ experiences, imagined experiences, persuasion from others as well as our physiological and emotional states (e.g., if a task makes us excited or nervous). Self-regulation, in turn, entails drawing on one’s confidence, making persistent goal-directed efforts, applying persistence, all of which are “more potent innate ability” (Maddux, 2011, p. 341); it also describes “the control that students have over their cognition, behavior, emotions and motivation through the use of personal strategies to achieve the goals they have established” (Panadero and Alonso-Tapia, 2014, p. 451). Importantly, Oxford (2017) links self-regulation to learner autonomy and agency: a sense of agency is required for self-regulatory behaviors (e.g., identifiable actions, such as making plans, monitoring the completion of smaller tasks, organizing one’s self and information or managing one’s learning environment) to emerge, and autonomous processes implement the choices the learner makes (either as an individual or in relation to others in their social environment). The combination of selves we develop comprises our self-concept: our perception of our physical, social, familiar, personal, academic, and other situational selves, self-evaluations and beliefs (including past, present and future selves). These selves emerge out of our experiences, both in isolation and influenced by comparisons to others. Related to self-concept is self-esteem, how we *evaluate* our self-image; in the writing process, this occurs often when students evaluate their work, which, as we have suggested in the introduction, is closely connected to their sense of self (Casanave, 2007). During learner-centered tasks, we can foster learners’ self-esteem in conjunction with their willingness to communicate, both by helping students see that they have something to contribute and, possibly, by generating ideas of how to express their “self”/“selves” in the target language through writing. Finally, self-verification is confirmation of our view of our self/selves, ideally mirrored by others, which helps us find coherence in our sense of self.

For the writing process and EMPATHICS, self-verification is apparent when we ask students to consider whether it is their own feedback or from others. We have addressed some issues connected to feedback above and would add the following as well: Having to address potential errors in writing can cause uncertainty for the student, whether it be related to grammatical features or content. We can mitigate these feelings by reassuring students that errors are inevitable in writing, even in their native language, and scaffold ways in which to identify and adjust errors. In fact, one of the many affordances of writing is its ability to facilitate reflection and revision, something that does not happen as readily in spoken expression (Byrnes and Manchón, 2014; Vasylets et al., 2017; Abrams, 2019).

Fortunately, ways of providing written feedback can be taught, and instructors can guide effective responses to that feedback. We suggest starting with some basic peer-editing techniques that help both the author and the editor maintain positive images of the self. Later, when these techniques become familiar, they can be applied to self-editing. Utilizing self-editing checklists and peer-editing activities before receiving faculty feedback (with potentially higher-stakes grading involved) can help foster self-efficacy, maintain self-esteem, and protect learners’ sense of self in general. In order to achieve these goals, however, it is paramount that the editing task is well-planned out, and that the participants be given a guide to support the learning task (Byrd, 2003). Even with a basic writing task, such as a brief dialogue, we can have students check for spelling errors or make certain that the register is correct. Beginning editing guides or checklists can be shown on a screen and we can model how to check for errors. As the writing tasks become more complex, we can add more or different details to check, dropping off ones that have been mastered. After a time, students can be encouraged to use the same techniques to review their own writing before turning it in. When creating editing tasks, it is important to remember that the goals of the lesson should be the focus of the editing activity (e.g., the use of cohesion devices, a clear narrative structure).

## Conclusion

In this paper, we examined teaching L2 writing with a new lens, connecting the process from pre-writing to editing and revisions to a model of positive psychology proposed by Oxford (2016). Accordingly, we suggested that writing tasks acknowledge learners negative emotions but emphasize positive ones in line with current research in positive psychology (Butler, 2019; Dewaele et al., 2019; Dewaele and Li, 2020; Fredrickson, 2004; MacIntyre and Gregersen, 2012; Oxford, 2016; Wang et al., 2021). This reframing can help learners find enjoyment in L2 writing tasks, increase their curiosity about the L2 and its related cultural contexts, build resilience, tap into resources stemming from multiple intelligences, and approach learning with empathy and perseverance. As language instructors, we might already be practicing many of the strategies described in this paper; we might already do pre-writing tasks and break larger projects into manageable steps, and we might already assign collaborative writing tasks or multimodal ones. However, becoming more aware of how negative and positive experiences might influence the outcome of writing instruction behooves us all. In addition to learning about the potential of positive psychology in the L2 classroom, and how it can be nurtured in L2 writing specifically, we need to make it a regular practice—a habit of pedagogy, if you will—to share this knowledge with the students explicitly, as they may not see it themselves.

## Author Contributions

ZsA and DB contributed equally to the preparation of this manuscript, from drafting to writing, all the way through the editing, and revision process. All authors contributed to the article and approved the submitted version.

## Conflict of Interest

The authors declare that the research was conducted in the absence of any commercial or financial relationships that could be construed as a potential conflict of interest.

## Publisher’s Note

All claims expressed in this article are solely those of the authors and do not necessarily represent those of their affiliated organizations, or those of the publisher, the editors and the reviewers. Any product that may be evaluated in this article, or claim that may be made by its manufacturer, is not guaranteed or endorsed by the publisher.

## Footnotes

^1^ https://www.thoughtco.com/rhetorical-stance-1692056
